# Methyl *N*-{(1*R*)-2-[(meth­oxy­carbon­yl)­oxy]-1-phenyleth­yl}carbamate

**DOI:** 10.1107/S2414314624002220

**Published:** 2024-03-21

**Authors:** María del Consuelo Mendoza Herrera, Mario Sampedro Cruz, Lydia María Pérez Díaz, José Antonio Rivera Márquez, Laura Orea Flores, Sylvain Bernès

**Affiliations:** a Facultad de Ingeniería Química, Benemérita Universidad Autónoma de Puebla, 72570 Puebla, Pue., Mexico; b Instituto de Ciencias, Benemérita Universidad Autónoma de Puebla, 72570 Puebla, Pue., Mexico; c Instituto de Física Luis Rivera Terrazas, Benemérita Universidad Autónoma de Puebla, 72570 Puebla, Pue., Mexico; University of Aberdeen, United Kingdom

**Keywords:** crystal structure, carbamate, phenyl­glycinol, supra­molecular chain, Sohncke group

## Abstract

The title mol­ecule, C_12_H_15_NO_5_, is a methyl carbamate derivative obtained by reacting (*R*)-2-phenyl­glycinol and methyl chloro­formate, with calcium hydroxide as heterogeneous catalyst. Supra­molecular chains are formed in the [100] direction, based on N—H⋯O hydrogen bonds between the amide and carboxyl­ate groups. These chains weakly inter­act in the crystal, and the phenyl rings do not display significant π–π inter­actions.

## Abstract 2

The chiral title compound forms supra­molecular chains, through N—H⋯O hydrogen bonds between the amide and carboxyl­ate groups.

## Graphical abstract

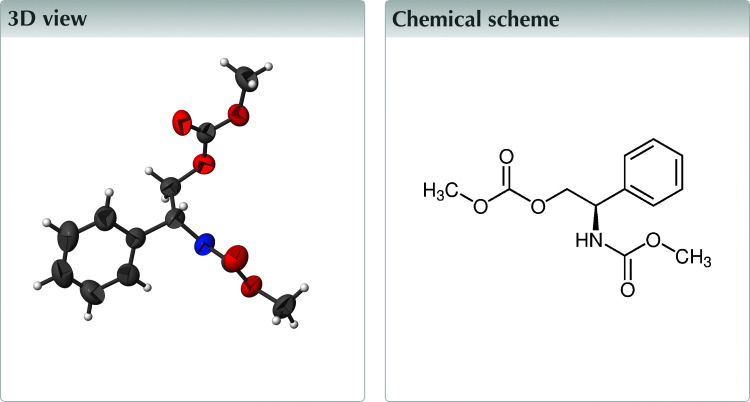

## Structure description

Methyl carbamate, Me(O)CONH_2_, the methyl ester of carbamic acid, is an important inter­mediate in the manufacture of carbamate-based resins used in the textile and polymer industries. On a smaller scale, it is also a pharmaceutical inter­mediate. The primary amine group can be functionalized, in the same way as for primary amides. From another point of view, the formation of a carbamate *via* a *N*-methyl­oxycarbonyl­ation reaction can also be considered as a useful protection of a primary amine (Sartori *et al.*, 2004 ▸). Finally, alternative routes allow both the formation of the carbamate and the *N*-functionalization. The title compound, C_12_H_15_NO_5_, resulted from such a reaction, between methyl chloro­formate and a chiral amino alcohol, namely (*R*)-2-phenyl­glycinol, under basic conditions, and using Ca(OH)_2_ as a heterogeneous catalyst.

**Figure 1:**
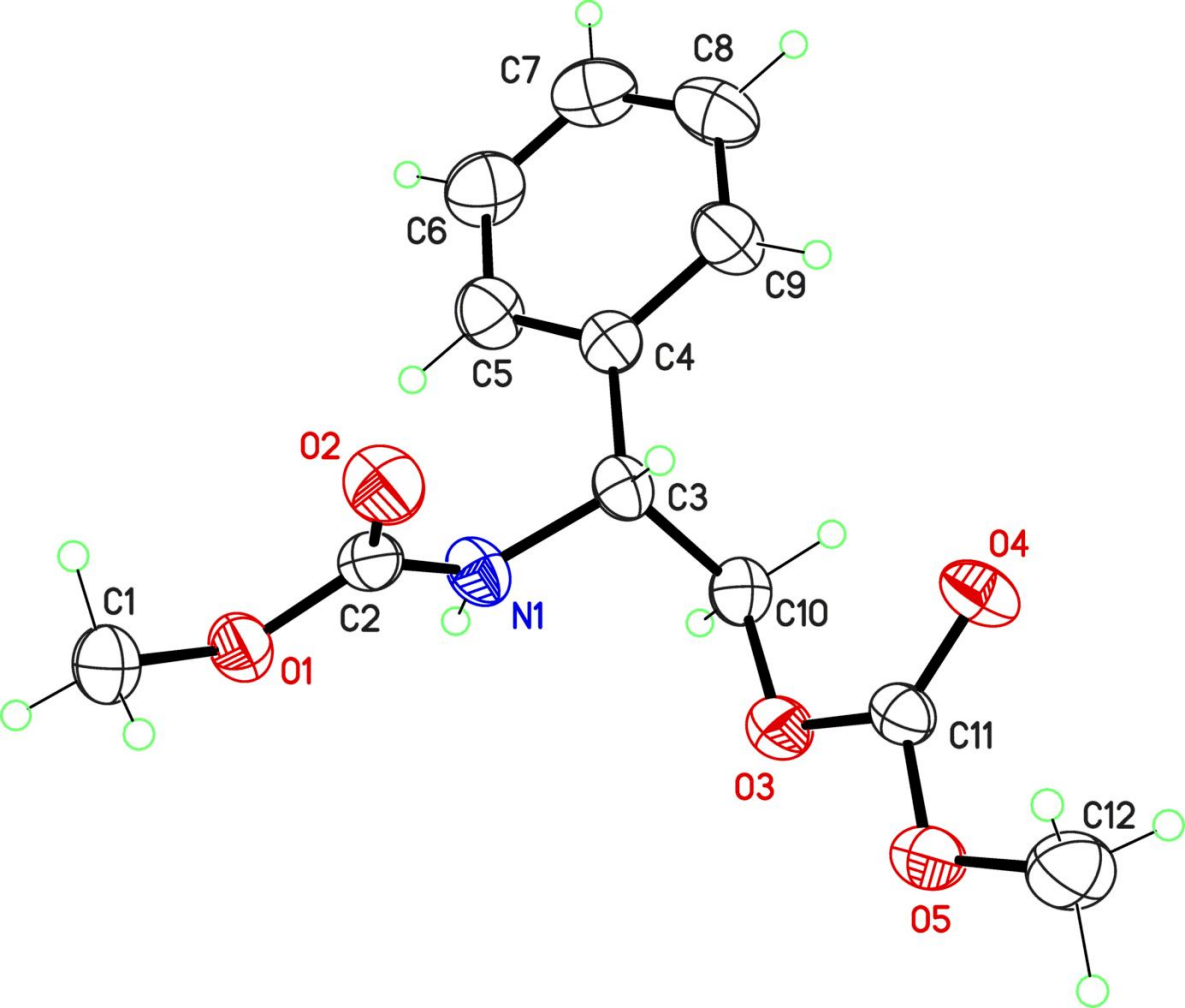
The mol­ecular structure of the title compound (50% probability ellipsoids).

We assumed that the *R* absolute configuration of the starting material was retained during the reaction, affording an enanti­omerically pure compound, which crystallized in the Sohncke space group *P*2_1_2_1_2_1_. Mol­ecular dimensions are as expected, and the amide group displays a geometry quite different from that of the carboxyl­ate group, with bond lengths C2—N1 = 1.333 (3) and C10—O3 = 1.445 (3) Å (Fig. 1 ▸). The geometry of the carbamate group is virtually identical to that observed in the closely related chiral compound meth­yl(1*S*-phenyl­eth­yl)carbamate, which crystallizes with four independent mol­ecules in the asymmetric unit (Thakar *et al.*, 2018 ▸).

**Figure 2:**
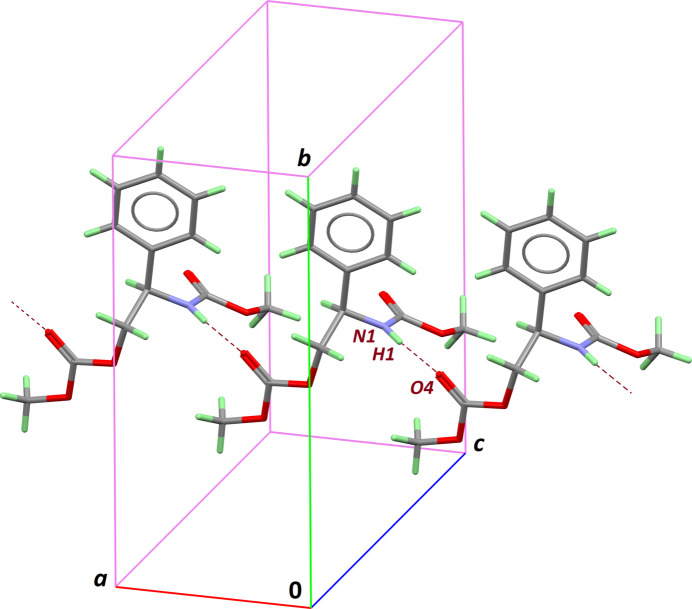
Supra­molecular chains formed in the [100] direction, based on amide–carboxyl­ate N—H⋯O hydrogen bonds (dashed bonds).

**Figure 3:**
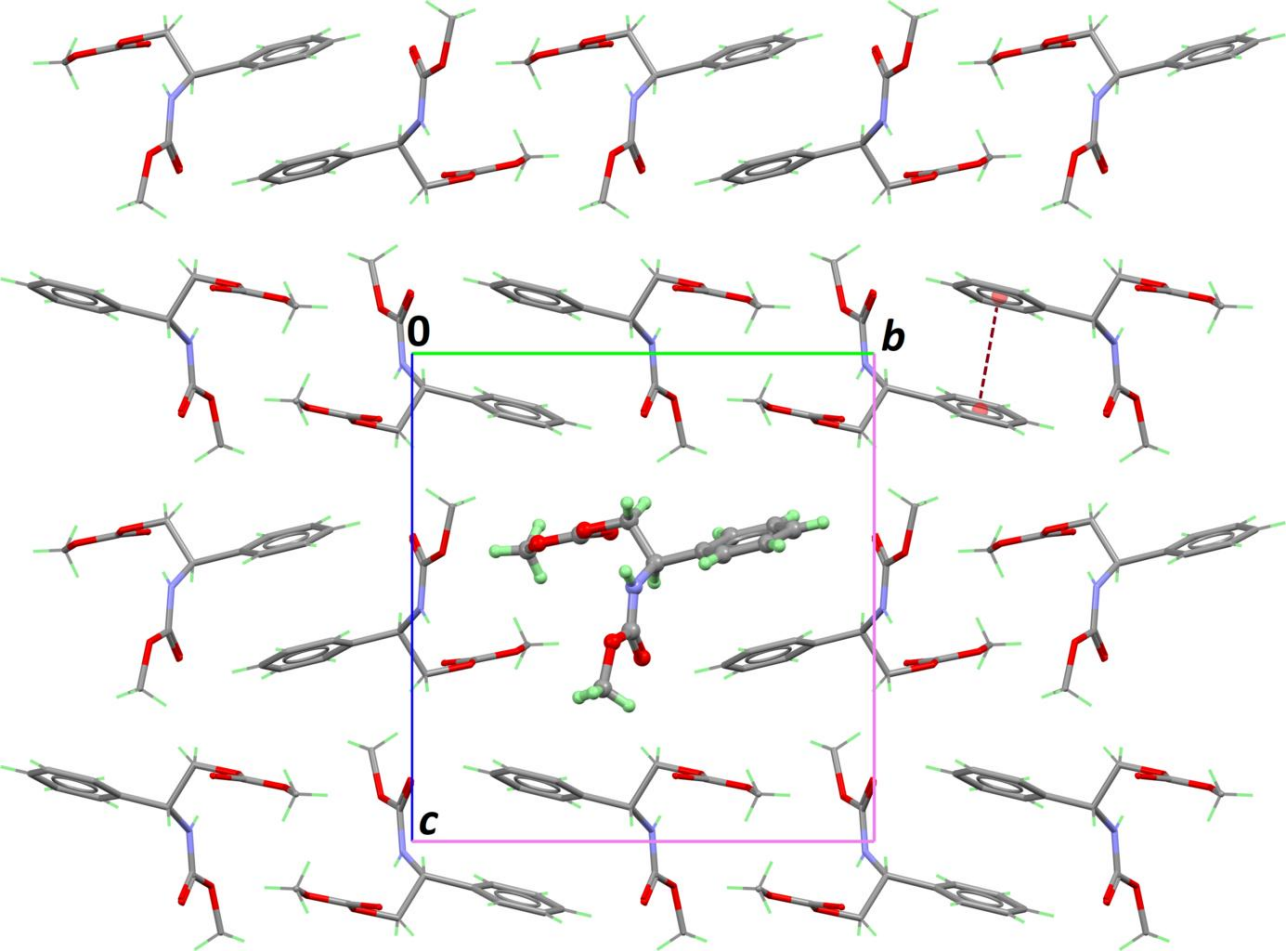
Part of the crystal structure of the title compound, viewed down the crystallographic *a* axis. The asymmetric unit is shown as a ball and stick model, and the dashed line represents the weak π–π inter­action between phenyl rings.

In the extended structure, the amide NH group serves as a donor, forming an inter­molecular hydrogen bond with the carboxyl­ate group C11=O4 of a neighbouring mol­ecule (Table 1 ▸). Infinite chains are then formed in the crystal, running along the short *a* axis (Fig. 2 ▸). Mol­ecules are further connected through weak C—H⋯O contacts involving the methyl group of the carbamate moiety as donor. Chains are arranged in the crystal with two neighbouring chains having the phenyl rings facing upwards (Fig. 3 ▸). However, no significant π–π contacts are observed: the distance separating two rings is large [4.6763 (17) Å] and the dihedral angle between corresponding mean planes is 21.84 (13)°. Aside from the weak C—H⋯O bonds mentioned above and van der Waals contacts, no other significant inter­actions between the supra­molecular chains are present in the crystal structure. As a consequence, the Kitaigorodskii packing index of 67.8% is rather low for this small organic mol­ecule (Spek, 2020 ▸).

## Synthesis and crystallization

(*R*)-2-Phenyl­glycinol (100 mg, 0.73 mmol) was dissolved in dry THF. The catalyst, Ca(OH)_2_ (10%), and methyl chloro­formate (0.56 ml, 7.2 mmol) were added, and the mixture was refluxed (333 K) under a nitro­gen atmosphere. After completion (TLC), the catalyst was separated by filtration, and the crude product recovered by elimination of the solvent under reduced pressure. The crude product was recrystallized from a mixture of solvents (hexa­ne:CH_2_Cl_*2*_, 4:1 *v*:*v*), affording single crystals suitable for X-ray diffraction. ^1^H-NMR (500 MHz, CDCl_3_): δ 3.66 (*s*, 3H), 3.76 (*s*, 3H), 4.35 (*s*, 2H), 5.05 (*broad*, 1H), 5.64 (*broad*, 1H), 7.29–7.37 (*m*, 5H) p.p.m. ^13^C-NMR (126 MHz, CDCl_3_): δ 52.33, 54.2, 55.06, 69.66, 126.59, 128.06, 128.80, 138.20, 155.68, 156.41 p.p.m.

## Refinement

Crystal data, data collection and structure refinement details are summarized in Table 2 ▸. The amide H atom (H1) was refined with free coordinates and isotropic displacement parameter. Other H atoms are in calculated positions. The absolute configuration was inferred from the synthesis.

## Supplementary Material

Crystal structure: contains datablock(s) I. DOI: 10.1107/S2414314624002220/hb4464sup1.cif

Structure factors: contains datablock(s) I. DOI: 10.1107/S2414314624002220/hb4464Isup2.hkl

Supporting information file. DOI: 10.1107/S2414314624002220/hb4464Isup3.cml

CCDC reference: 2338571

Additional supporting information:  crystallographic information; 3D view; checkCIF report

## Figures and Tables

**Table 1:** Hydrogen-bond geometry (Å, °)

*D*—H⋯*A*	*D*—H	H⋯*A*	*D*⋯*A*	*D*—H⋯*A*
N1—H1⋯O4^i^	0.82 (3)	2.10 (3)	2.917 (2)	175 (2)
C1—H1*B*⋯O4^ii^	0.96	2.59	3.494 (3)	158
C1—H1*C*⋯O5^iii^	0.96	2.62	3.332 (4)	131

Symmetry codes: (i) 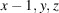 ; (ii) 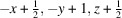 ; (iii) 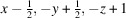 .

**Table 2:** Experimental details

Crystal data	
Chemical formula	C_12_H_15_NO_5_
*M*_r_	253.25
Crystal system, space group	Orthorhombic, *P*2_1_2_1_2_1_
Temperature (K)	295
*a*, *b*, *c* (Å)	6.2497 (2), 13.8633 (6), 14.6254 (5)
*V* (Å^3^)	1267.17 (8)
*Z*	4
Radiation type	Mo *K*α
μ (mm^−1^)	0.10
Crystal size (mm)	0.67 × 0.29 × 0.16

Data collection	
Diffractometer	Xcalibur, Atlas, Gemini
Absorption correction	Multi-scan (*CrysAlis PRO*; Rigaku OD, 2022 ▸)
*T*_min_, *T*_max_	0.967, 1.000
No. of measured, independent and observed [*I* > 2σ(*I*)] reflections	24119, 3859, 2841
*R*_int_	0.035
(sin θ/λ)_max_ (Å^−1^)	0.714

Refinement	
*R*[*F* ^2^ > 2σ(*F* ^2^)], *wR*(*F* ^2^), *S*	0.046, 0.118, 1.03
No. of reflections	3859
No. of parameters	169
H-atom treatment	H atoms treated by a mixture of independent and constrained refinement
Δρ_max_, Δρ_min_ (e Å^−3^)	0.14, −0.16

Computer programs: *CrysAlis PRO* (Rigaku OD, 2022 ▸), *SHELXT2018/2* (Sheldrick, 2015*a* ▸), *SHELXL2018/3* (Sheldrick, 2015*b* ▸), *XP* in *SHELXTL-Plus* (Sheldrick, 2008 ▸), *Mercury* (Macrae *et al.*, 2020 ▸) and *publCIF* (Westrip, 2010 ▸).

## full crystallographic data

### Crystal data

**Table:**

C_12_H_15_NO_5_	*D*_x_ = 1.327 Mg m^−^^3^
*M**_r_* = 253.25	Melting point: 345 K
Orthorhombic, *P*2_1_2_1_2_1_	Mo *K*α radiation, λ = 0.71073 Å
*a* = 6.2497 (2) Å	Cell parameters from 4907 reflections
*b* = 13.8633 (6) Å	θ = 3.3–26.1°
*c* = 14.6254 (5) Å	µ = 0.10 mm^−^^1^
*V* = 1267.17 (8) Å^3^	*T* = 295 K
*Z* = 4	Block, colourless
*F*(000) = 536	0.67 × 0.29 × 0.16 mm

### Data collection

**Table:**

Xcalibur, Atlas, Gemini diffractometer	3859 independent reflections
Radiation source: fine-focus sealed X-ray tube, Enhance (Mo) X-ray Source	2841 reflections with *I* > 2σ(*I*)
Graphite monochromator	*R*_int_ = 0.035
Detector resolution: 10.5564 pixels mm^-1^	θ_max_ = 30.5°, θ_min_ = 2.9°
ω scans	*h* = −8→8
Absorption correction: multi-scan (CrysAlisPro; Rigaku OD, 2022)	*k* = −19→19
*T*_min_ = 0.967, *T*_max_ = 1.000	*l* = −20→20
24119 measured reflections	

### Refinement

**Table:**

Refinement on *F*^2^	Primary atom site location: dual
Least-squares matrix: full	Secondary atom site location: difference Fourier map
*R*[*F*^2^ > 2σ(*F*^2^)] = 0.046	Hydrogen site location: mixed
*wR*(*F*^2^) = 0.118	H atoms treated by a mixture of independent and constrained refinement
*S* = 1.03	*w* = 1/[σ^2^(*F*_o_^2^) + (0.0587*P*)^2^ + 0.052*P*] where *P* = (*F*_o_^2^ + 2*F*_c_^2^)/3
3859 reflections	(Δ/σ)_max_ < 0.001
169 parameters	Δρ_max_ = 0.14 e Å^−^^3^
0 restraints	Δρ_min_ = −0.16 e Å^−^^3^
0 constraints	

### Fractional atomic coordinates and isotropic or equivalent isotropic displacement parameters (Å^2^)

**Table:**

	*x*	*y*	*z*	*U*_iso_*/*U*_eq_	
O1	−0.2085 (3)	0.43365 (12)	0.59140 (10)	0.0598 (4)	
O2	0.1160 (3)	0.49818 (13)	0.62348 (10)	0.0670 (5)	
O3	0.2905 (2)	0.37368 (12)	0.36207 (12)	0.0607 (4)	
O4	0.6279 (2)	0.42897 (11)	0.36564 (11)	0.0597 (4)	
O5	0.5449 (3)	0.27357 (12)	0.38930 (13)	0.0668 (5)	
N1	−0.0043 (3)	0.48073 (14)	0.47814 (11)	0.0527 (4)	
H1	−0.105 (4)	0.4625 (18)	0.4474 (16)	0.053 (6)*	
C1	−0.2419 (5)	0.4127 (2)	0.68630 (17)	0.0769 (8)	
H1A	−0.387398	0.392701	0.695569	0.115*	
H1B	−0.213777	0.469417	0.721969	0.115*	
H1C	−0.147035	0.361905	0.704931	0.115*	
C2	−0.0183 (3)	0.47379 (15)	0.56885 (13)	0.0475 (4)	
C3	0.1699 (3)	0.52669 (16)	0.43010 (13)	0.0473 (5)	
H3	0.297760	0.524175	0.468833	0.057*	
C4	0.1259 (4)	0.63165 (16)	0.40547 (13)	0.0492 (5)	
C5	−0.0757 (4)	0.6723 (2)	0.41155 (19)	0.0678 (7)	
H5	−0.190104	0.634538	0.430815	0.081*	
C6	−0.1102 (5)	0.7682 (2)	0.3895 (2)	0.0834 (8)	
H6	−0.246934	0.794183	0.394150	0.100*	
C7	0.0544 (6)	0.8242 (2)	0.36108 (19)	0.0790 (8)	
H7	0.030618	0.888693	0.346652	0.095*	
C8	0.2556 (6)	0.7862 (2)	0.3536 (2)	0.0868 (9)	
H8	0.368411	0.824713	0.333876	0.104*	
C9	0.2916 (5)	0.6898 (2)	0.37548 (19)	0.0725 (7)	
H9	0.428547	0.664215	0.369869	0.087*	
C10	0.2142 (4)	0.46992 (18)	0.34271 (14)	0.0541 (5)	
H10A	0.083947	0.465903	0.306877	0.065*	
H10B	0.320390	0.503898	0.306596	0.065*	
C11	0.5013 (4)	0.36477 (15)	0.37178 (13)	0.0492 (4)	
C12	0.7676 (4)	0.2503 (2)	0.4027 (2)	0.0787 (8)	
H12A	0.850042	0.275381	0.352705	0.118*	
H12B	0.784356	0.181520	0.405485	0.118*	
H12C	0.816598	0.278478	0.458896	0.118*	

### Atomic displacement parameters (Å^2^)

**Table:**

	*U*^11^	*U*^22^	*U*^33^	*U*^12^	*U*^13^	*U*^23^
O1	0.0627 (9)	0.0623 (9)	0.0543 (8)	−0.0127 (8)	0.0094 (7)	−0.0026 (7)
O2	0.0727 (10)	0.0791 (12)	0.0492 (8)	−0.0157 (9)	−0.0091 (8)	−0.0040 (8)
O3	0.0484 (8)	0.0545 (9)	0.0791 (10)	−0.0078 (7)	0.0020 (8)	−0.0061 (8)
O4	0.0521 (8)	0.0539 (9)	0.0732 (10)	−0.0146 (7)	−0.0156 (8)	0.0141 (8)
O5	0.0597 (9)	0.0467 (9)	0.0940 (12)	−0.0059 (7)	0.0045 (8)	0.0026 (8)
N1	0.0464 (9)	0.0661 (11)	0.0457 (9)	−0.0137 (9)	−0.0059 (8)	0.0020 (8)
C1	0.098 (2)	0.0742 (19)	0.0583 (13)	−0.0119 (15)	0.0262 (14)	−0.0041 (12)
C2	0.0533 (11)	0.0412 (10)	0.0479 (10)	0.0005 (9)	−0.0004 (9)	−0.0013 (8)
C3	0.0395 (9)	0.0585 (12)	0.0439 (9)	−0.0055 (9)	−0.0062 (7)	0.0019 (8)
C4	0.0512 (11)	0.0560 (12)	0.0403 (9)	−0.0038 (10)	−0.0048 (9)	−0.0002 (9)
C5	0.0576 (14)	0.0716 (17)	0.0742 (15)	0.0022 (12)	−0.0028 (11)	0.0071 (14)
C6	0.0823 (19)	0.0727 (19)	0.095 (2)	0.0178 (16)	−0.0093 (17)	0.0056 (15)
C7	0.107 (3)	0.0602 (15)	0.0697 (16)	0.0062 (16)	−0.0161 (15)	0.0083 (13)
C8	0.102 (2)	0.069 (2)	0.0893 (19)	−0.0242 (17)	−0.0026 (18)	0.0189 (15)
C9	0.0614 (14)	0.0686 (16)	0.0873 (17)	−0.0081 (12)	0.0014 (14)	0.0136 (14)
C10	0.0488 (10)	0.0626 (14)	0.0508 (10)	0.0009 (10)	−0.0021 (9)	0.0004 (10)
C11	0.0524 (11)	0.0489 (11)	0.0464 (10)	−0.0090 (10)	−0.0008 (10)	−0.0009 (9)
C12	0.0703 (16)	0.0606 (16)	0.105 (2)	0.0071 (12)	−0.0097 (17)	0.0120 (15)

### Geometric parameters (Å, º)

**Table:**

O1—C2	1.353 (3)	C4—C5	1.383 (3)
O1—C1	1.433 (3)	C4—C9	1.384 (3)
O2—C2	1.207 (2)	C5—C6	1.385 (4)
O3—C11	1.331 (3)	C5—H5	0.9300
O3—C10	1.445 (3)	C6—C7	1.355 (4)
O4—C11	1.194 (2)	C6—H6	0.9300
O5—C11	1.318 (3)	C7—C8	1.368 (5)
O5—C12	1.442 (3)	C7—H7	0.9300
N1—C2	1.333 (3)	C8—C9	1.392 (4)
N1—C3	1.444 (3)	C8—H8	0.9300
N1—H1	0.82 (3)	C9—H9	0.9300
C1—H1A	0.9600	C10—H10A	0.9700
C1—H1B	0.9600	C10—H10B	0.9700
C1—H1C	0.9600	C12—H12A	0.9600
C3—C4	1.524 (3)	C12—H12B	0.9600
C3—C10	1.526 (3)	C12—H12C	0.9600
C3—H3	0.9800		
			
C2—O1—C1	116.6 (2)	C7—C6—C5	120.3 (3)
C11—O3—C10	115.66 (17)	C7—C6—H6	119.9
C11—O5—C12	116.11 (19)	C5—C6—H6	119.9
C2—N1—C3	124.45 (18)	C6—C7—C8	120.1 (3)
C2—N1—H1	118.4 (17)	C6—C7—H7	120.0
C3—N1—H1	116.9 (17)	C8—C7—H7	120.0
O1—C1—H1A	109.5	C7—C8—C9	120.0 (3)
O1—C1—H1B	109.5	C7—C8—H8	120.0
H1A—C1—H1B	109.5	C9—C8—H8	120.0
O1—C1—H1C	109.5	C4—C9—C8	120.7 (3)
H1A—C1—H1C	109.5	C4—C9—H9	119.6
H1B—C1—H1C	109.5	C8—C9—H9	119.6
O2—C2—N1	126.4 (2)	O3—C10—C3	111.83 (17)
O2—C2—O1	124.37 (19)	O3—C10—H10A	109.2
N1—C2—O1	109.27 (18)	C3—C10—H10A	109.2
N1—C3—C4	113.60 (18)	O3—C10—H10B	109.2
N1—C3—C10	108.46 (18)	C3—C10—H10B	109.2
C4—C3—C10	109.09 (17)	H10A—C10—H10B	107.9
N1—C3—H3	108.5	O4—C11—O5	126.4 (2)
C4—C3—H3	108.5	O4—C11—O3	125.3 (2)
C10—C3—H3	108.5	O5—C11—O3	108.30 (18)
C5—C4—C9	117.7 (2)	O5—C12—H12A	109.5
C5—C4—C3	122.6 (2)	O5—C12—H12B	109.5
C9—C4—C3	119.8 (2)	H12A—C12—H12B	109.5
C4—C5—C6	121.2 (3)	O5—C12—H12C	109.5
C4—C5—H5	119.4	H12A—C12—H12C	109.5
C6—C5—H5	119.4	H12B—C12—H12C	109.5
			
C3—N1—C2—O2	−5.4 (4)	C5—C6—C7—C8	0.4 (5)
C3—N1—C2—O1	175.16 (19)	C6—C7—C8—C9	−0.2 (5)
C1—O1—C2—O2	−5.0 (3)	C5—C4—C9—C8	0.9 (4)
C1—O1—C2—N1	174.4 (2)	C3—C4—C9—C8	−179.3 (2)
C2—N1—C3—C4	−95.0 (2)	C7—C8—C9—C4	−0.4 (5)
C2—N1—C3—C10	143.5 (2)	C11—O3—C10—C3	−88.4 (2)
N1—C3—C4—C5	−12.6 (3)	N1—C3—C10—O3	−65.0 (2)
C10—C3—C4—C5	108.5 (2)	C4—C3—C10—O3	170.79 (16)
N1—C3—C4—C9	167.6 (2)	C12—O5—C11—O4	0.4 (4)
C10—C3—C4—C9	−71.3 (3)	C12—O5—C11—O3	−179.5 (2)
C9—C4—C5—C6	−0.8 (4)	C10—O3—C11—O4	−0.1 (3)
C3—C4—C5—C6	179.4 (2)	C10—O3—C11—O5	179.81 (17)
C4—C5—C6—C7	0.2 (5)		

### Hydrogen-bond geometry (Å, º)

**Table:**

*D*—H···*A*	*D*—H	H···*A*	*D*···*A*	*D*—H···*A*
N1—H1···O4^i^	0.82 (3)	2.10 (3)	2.917 (2)	175 (2)
C1—H1*B*···O4^ii^	0.96	2.59	3.494 (3)	158
C1—H1*C*···O5^iii^	0.96	2.62	3.332 (4)	131

Symmetry codes: (i) *x*−1, *y*, *z*; (ii) −*x*+1/2, −*y*+1, *z*+1/2; (iii) *x*−1/2, −*y*+1/2, −*z*+1.
